# The distribution of plant consumption traits across habitat types and the patterns of fruit availability suggest a mechanism of coexistence of two sympatric frugivorous mammals

**DOI:** 10.1002/ece3.5017

**Published:** 2019-04-01

**Authors:** Luc Roscelin Dongmo Tédonzong, Jacob Willie, Nikki Tagg, Martin N. Tchamba, Tsi Evaristus Angwafo, Ada Myriane Patipe Keuko, Jacques Keumo Kuenbou, Charles‐Albert Petre, Luc Lens

**Affiliations:** ^1^ Projet Grands Singes (PGS), Cameroun, Centre for Research and Conservation (CRC) Royal Zoological Society of Antwerpen (RZSA) Antwerpen Belgium; ^2^ Terrestrial Ecology Unit (TEREC), Department of Biology Ghent University (UGent) Ghent Belgium; ^3^ Department of Forestry University of Dschang Dschang Cameroon; ^4^ Department of Fundamental Sciences The University of Bamenda, HTTTC Bambili Cameroon; ^5^ Laboratory of Tropical Forestry University of Liège Gembloux Agro‐Bio Tech Liège Belgium; ^6^ Conservation Biology Unit, Directorate Natural Environment Royal Belgian Institute of Natural Sciences Brussels Belgium

**Keywords:** ecological niche, fallback food, fruit phenology, fruit preference, habitat selection, Niche partitioning in primates

## Abstract

Understanding the mechanisms governing the coexistence of organisms is an important question in ecology, and providing potential solutions contributes to conservation science. In this study, we evaluated the contribution of several mechanisms to the coexistence of two sympatric frugivores, using western lowland gorillas (*Gorilla gorilla gorilla*) and central chimpanzees (*Pan troglodytes troglodytes*) in a tropical rainforest of southeast Cameroon as a model system. We collected great ape fecal samples to determine and classify fruit species consumed; we conducted great ape nest surveys to evaluate seasonal patterns of habitat use; and we collected botanical data to investigate the distribution of plant species across habitat types in relation to their “consumption traits” (which indicate whether plants are preferred or fallback for either gorilla, chimpanzee, or both). We found that patterns of habitat use varied seasonally for both gorillas and chimpanzees and that gorilla and chimpanzee preferred and fallback fruits differed. Also, the distribution of plant consumption traits was influenced by habitat type and matched accordingly with the patterns of habitat use by gorillas and chimpanzees. We show that neither habitat selection nor fruit preference alone can explain the coexistence of gorillas and chimpanzees, but that considering together the distribution of plant consumption traits of fruiting woody plants across habitats as well as the pattern of fruit availability may contribute to explaining coexistence. This supports the assumptions of niche theory with dominant and subordinate species in heterogeneous landscapes, whereby a species may prefer nesting in habitats where it is less subject to competitive exclusion and where food availability is higher. To our knowledge, our study is the first to investigate the contribution of plant consumption traits, seasonality, and habitat heterogeneity to enabling the coexistence of two sympatric frugivores.

**OPEN RESEARCH BADGES:**

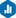

This article has earned an Open Data Badge for making publicly available the digitally‐shareable data necessary to reproduce the reported results. The data is available at https://datadryad.org/resource/doi:10.5061/dryad.ms65f29.

## 1 INTRODUCTION

A great challenge in ecology is understanding the evolutionary and ecological implications of biotic interactions (Sutherland et al., 2013), which has led researchers to question the mechanisms shaping the coexistence of closely related species (Benítez‐López, Viñuela, Suárez, Hervás, & García, 2014; Hutchinson, 1961; Kotler & Brown, 2007). Many factors have been reported to control space use by animal species, including abiotic factors, such as climatic variables, and biotic factors, such as resource availability, resource overlap between two species and their relative positions in the food web (Kotler & Brown, 2007). The necessity to consider species interactions in species distribution models has advanced our understanding of how overlap in resource use and particular characteristics of those resources influences coexistence of two species (Benítez‐López et al., 2014; Leach, Montgomery, & Reid, 2016).

Resources within an animal's niche can be entirely available to them if no competitor or predator is present and is thus referred to as a “fundamental niche” (Hutchinson, 1957). However, the presence of competitors prevents the use of the entire fundamental niche, restricting the organism to its “realized niche,” which is a subset of its fundamental niche (Hutchinson, 1957). The niche overlap between two closely related species is a fundamental aspect of “niche theory,” which states that two closely related species occupying the same niche may undergo competition (Pianka, 1981). Competition may be direct (dealing with space), called “interference competition,” or indirect (dealing with resources), called “exploitation competition” (Pianka, 1981). High levels of competition may lead to the competitive exclusion of one species by the most dominant competitor (Hutchinson, 1961) and are thus not consistent with species coexistence. Importantly, species may have become specialized through character displacement (e.g., morphological differentiation) by partitioning the shared resource (Walter, 1991). Such niche partitioning reduces exploitation competition (Rosenzweig, 1981), leading to a divergence of realized niches (Sinclair, Fryxell, & Caughley, 2006; Walter, 1991) and facilitating coexistence. This implies that the sympatric species may have reached some equilibrium in the use of resources that allow them to coexist (Pianka, 1981). Spatial or temporal variations in resource availability can lead to changes in the pattern of habitat use by sympatric species (Grether, Losin, Anderson, & Okamoto, 2009; Rosenzweig, 1981).

Studying a single species can help understand ranging patterns, but integrating the study of biotic interactions between two closely related species can inform on how their abundance and distribution may be influenced by their niche breadth or niche position (Benítez‐López et al., 2014; Gaston, Blackburn, & Lawton, 1997). Many aspects, such as reproductive success and resource use, are important to the niche theory, but of particular interest in understanding species interactions is the pattern of resource use (including food and space) (Pianka, 1981). Although shared resources are central to the concept of interspecific competition, the limited nature of resources is the ultimate cause of competition (Amarasekare, 2003). Resource supplies can be continuously reduced, but reduction can also occur on a temporal basis, leading to temporal niche differentiation between species (Hutchinson, 1961). In this case, understanding species interaction is only possible by analyzing the various ways in which different resources are used by different species across time (Amarasekare, 2003).

Two hypotheses have been proposed to explain patterns of resource use by two coexisting species, namely the “optimal foraging theory” and the “habitat selection theory” (Rosenzweig, 1981). Both hypotheses have gained support from studies investigating the role of resource quality in niche partitioning (Gregory & Gaston, 2000; Kamilar & Ledogar, 2011; Kotler & Brown, 1988; de Longh et al., 2011; Martin & Garnett, 2013; Oelze, Head, Robbins, Richards, & Boesch, 2014; Perrin & Kotler, 2005; Ritchie, 2002). Which hypothesis gains support depends upon how species alter their use of preferred resources in cases of high and low availability (Rosenzweig, 1981). Different feeding plant resources contain different nutritional compositions that make them either preferred or fallback for animal species (Doran‐Sheehy, Mongo, Lodwick, & Conklin‐Brittain, 2009; Remis, Dierenfeld, Mowry, & Carroll, 2001), and their diversity and community structure are the resultant habitat heterogeneity (Myers & Harms, 2009). This implies that the availability of different food types may vary with habitat types and seasons due to phenological patterns in plants (Poulsen & Clark, 2004; Yamagiwa, Basabose, Kaleme, & Yumoto, 2008). However, the distribution of such resources, such as different food types (preferred and fallback) as an indication of food quality for the animal species, has rarely been considered when investigating mechanisms that may facilitate the coexistence of two closely related species in heterogeneous environments. Preferred foods are consumed whenever they are available, while fallback foods are consumed when preferred foods are scarce (Yamagiwa & Basabose, 2009). A few studies have considered the abundance of resources (Brown, 1989; Kotler & Brown, 1988; Steinmetz, Garshelis, Chutipong, & Seuaturien, 2013) or their diversity (Kleynhans, Jolles, Bos, & Olff, 2011; Kotler & Brown, 1988), but without taking into account the intrinsic value of each resource to the animal consumers, or considering food resource quality at the community level (Owen‐Smith, Martin, & Yoganand, 2015; Steinmetz et al., 2013). A study by Vélez, Espelta, Rivera, and Armenteras (2017) investigated how the distribution of preferred fruits influenced habitat use by lowland tapirs (*Tapirus terrestris*), but did not evaluate the implications for coexistence with closely related species.

We aimed to evaluate whether varying spatial and temporal availability of fruiting woody plant resources (comprising trees and lianas) can contribute to explaining the coexistence of two sympatric frugivores. Coexistence of two frugivorous species depends upon the outcome of species competition, which is in turn dependent upon the superiority and inferiority of the competitors (Perrin & Kotler, 2005). The superior competitors may be characterized as the species that rely the most on fruit (preferred fruits), while the inferior competitors tend to be more folivorous (Kinahan & Pillay, 2008), increasing their consumption of vegetation and lower quality fruits (fallback fruits) to reduce the interspecific competition when fruit availability is low (Kinahan & Pillay, 2008; Martin & Garnett, 2013).

We used sympatric great apes (western lowland gorillas *Gorilla gorilla gorilla* and central chimpanzees *Pan troglodytes troglodytes*) as model species, because they occur in the same habitats, share feeding habits to some extent (Tutin, Fernandez, Rogers, Williamson, & Mcgrew, 1991), and the availability of their preferred food (fruits) varies seasonally (Tweheyo & Lye, 2003; Yamagiwa et al., 2008). Fruit constitutes an important part of gorilla and chimpanzee diet with consequences in their ranging patterns (Doran‐Sheehy, Greer, Mongo, & Schwindt, 2004), and the two species exhibit a high level of dietary overlap in terms of number of species (Tutin et al., 1991), and more than 93% of their fruits are obtained from woody plant species (Tutin & Fernandez, 1993). When fruit availability is high, both gorillas and chimpanzees increase their fruit consumption, but when fruit availability is low, chimpanzees maintain a diet dominated by fruit while gorillas incorporate large quantities of vegetative foods (herbs, leaves, flowers) (Basabose & Yamagiwa, 2002; Oelze et al., 2014). Fruits (preferred and fallback) are arguably the most influential aspects of great ape ecology (Lambert & Rothman, 2015; Poulsen & Clark, 2004) and represent the main cause of interspecific competitive interactions in primates (Yamagiwa, Maruhashi, Yumoto, & Mwanza, 1996). Additionally, studies have shown that chimpanzees prefer nesting in closed old growth forests, while gorillas prefer nesting in open young forests, as well as swamps and light gaps (Morgan, Sanz, Onononga, & Strindberg, 2006; Willie, Petre, Tagg, & Lens, 2013). Therefore, we propose that chimpanzees may have a higher competitive ability and be more specialized in fruit consumption than gorillas.

We tested two mechanisms that may promote the coexistence of gorillas and chimpanzees, described by the main niche axes: diet breadth and habitat selection. As fruiting woody plant species are situated within habitat types, we tested a third mechanism described by a combination of the first two and defined by the spatial variation in the fruiting woody plants across the different habitat types based on whether they are preferred or fallback for either gorillas, chimpanzees or both, hereafter termed “plant consumption traits.” Consumption traits describe the quality of the fruiting species to the animal: high‐quality fruits being “preferred” and lower quality fruits, consumed when high‐quality fruits are unavailable, being “fallback.” We asked whether differences in habitat selection and fruit preference and the distribution of fruiting woody plants can help explain the coexistence of gorillas and chimpanzees. We hypothesized that (a) if habitat selection alone is the underlying mechanism of coexistence of gorillas and chimpanzees, the same pattern of habitat selection must be observed across all seasons; (b) if differential diet breadth alone is responsible for the coexistence of gorillas and chimpanzees, the pattern of habitat selection will not be different between the two species across all seasons; (c) the distribution of preferred and fallback woody fruiting plant species across habitat types may explain the coexistence of gorillas and chimpanzees; the seasonal availability of preferred and fallback fruits may vary between habitats, and the seasonal patterns of habitat use may vary between species. For this third hypothesis, considering chimpanzees as the superior competitor, we predicted that preferred species for both animals would be more associated with chimpanzee commonly preferred habitats (old secondary forests [Arnhem, Dupain, Drubbel, Devos, & Vercauteren, 2008; Tédonzong et al., 2018]); while fallback species would be more associated with gorilla commonly preferred habitats (young secondary forests, opened forests and swamps [Willie et al., 2013]). Also, gorillas may avoid nesting in habitats preferred by chimpanzees to escape competitive exclusion but may still forage in those chimpanzees‐preferred nesting habitats when their preferred fruits are available there.

## 2 METHODOLOGY

### 2.1 Study area

**Figure 1:**
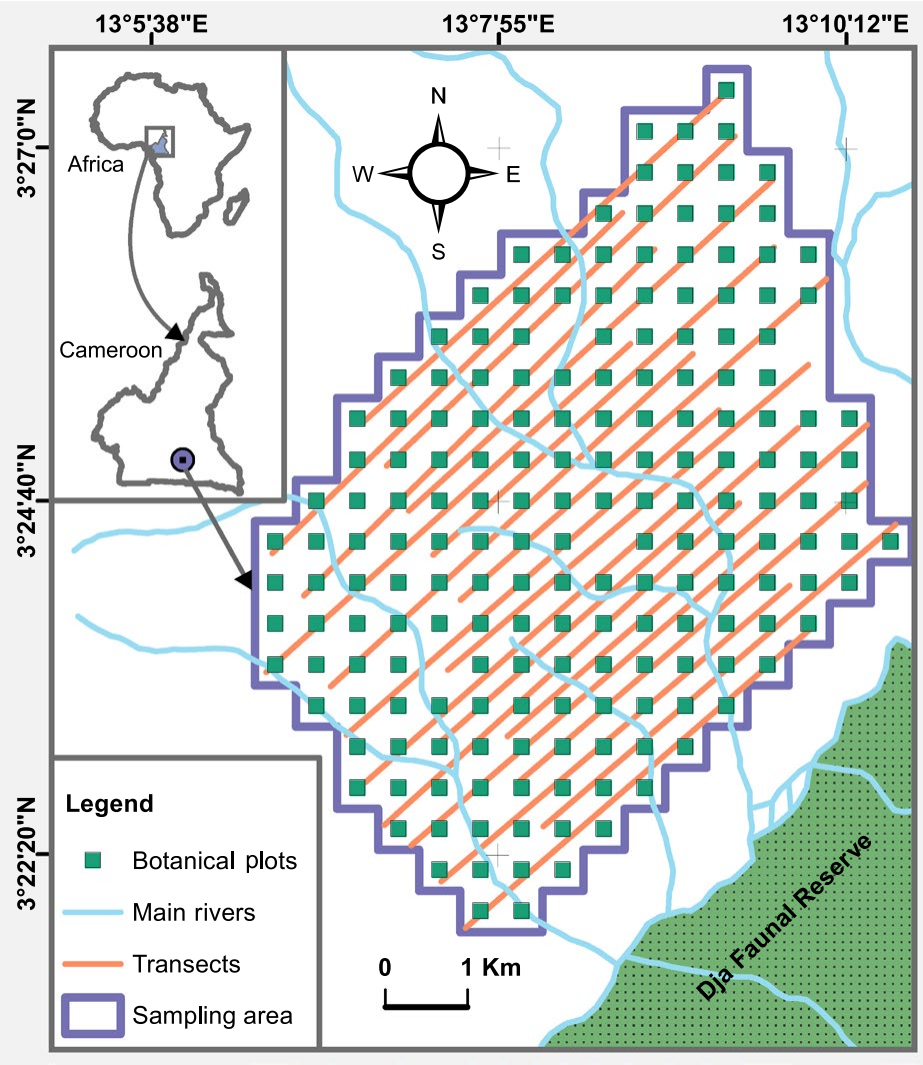
Study area and sampling design

We conducted this study in the research site “La Belgique,” located in the Forest Management Unit 10 047a, at the northern periphery of the Dja Faunal Reserve, Cameroon, located between 13°5′E and 13°11′E, and 3°21′N and 3°28′N (Figure 1). The mean elevation of the site is 680.58 m (*SD* = 17.53 m, range: 633–751 m) (Tédonzong et al., 2018). Climate data indicate two dry seasons and two rainy seasons: the long dry season lasts from November to February and the short dry season from July to August; while the long rainy season extends February to July and the short rainy season August to November (Willie, Tagg, Petre, Pereboom, & Lens, 2014). Rainfall is an average of 1637.9 mm per year (*SD* = 105.1 mm) and temperature averages range from 19.5°C (*SD* = 1.3°) to 26.3°C (*SD* = 2.4°C) (Willie et al., 2014).

**Figure A1:**
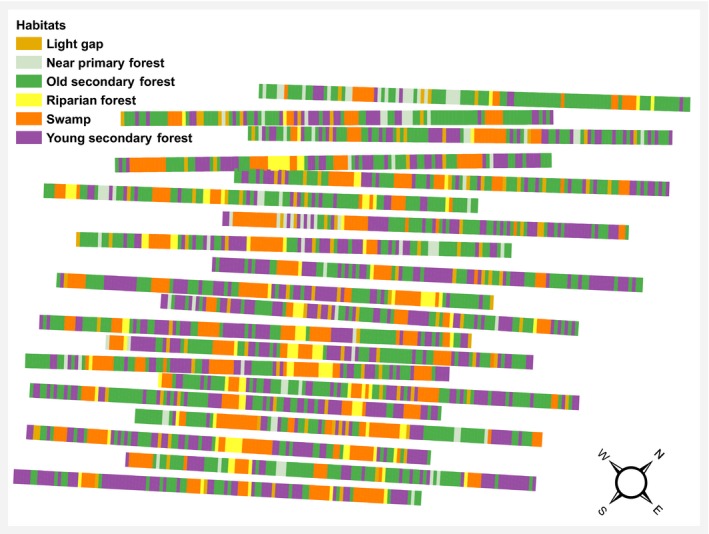
Distribution of the different habitat types along the transects

We considered five habitat types in our study, based on the physical structure of the forest, the height of the dominant trees, and the hydromorphic status of the soil: Mature Forests (MF), Young Secondary Forests (YSF), Light Gaps (LG), Swamps (SW) and Riparian Forests (RF), modified from Willie et al. (2013), Willie et al. (2014) (Figure A1). We decided to combine the categories Near Primary Forests (NPF) and Old Secondary Forests (OSF), as defined by Willie et al. (2013), Willie et al. (2014), into MF, as NPF are under‐represented in our study site (<5%) and both NPF and OSF represent forests at advanced levels of stand development (Willie et al., 2013). Following Willie et al. (2013), Willie et al. (2014), MF are characterized by the presence of large, tall trees with diameter at breast height (DBH) >60 cm and height >25 m, and of tree species such as *Piptadeniastrum africanum* (Mimosaceae) and *Distemonanthus benthamianus* (Ceasalpiniaceae). YSF are characterized by trees of smaller DBH and height (<25 cm and <25 m, respectively) than MF. YSF understory is very dense, with the presence of pioneer trees (e.g., species such as *Tabernaemontana crassa* [Apocynaceae]). SW are permanently flooded areas characterized by the dominance of *Raphia *spp. RF are only temporally flooded, occurring at the interface between terra firma habitat types (MF and YSF) and swamps, and consequently sharing many terra firma and swamp species. LG are created because of tree falls (due to elephant activity, wind or natural death) and can then appear in any other habitat type described above, generally on small spots. However, they represent microhabitats that are known to be favored by gorillas for nesting (Willie et al., 2013).

### 2.2 Data collection

#### 2.2.1 Great ape and habitat surveys

We conducted great ape nest surveys on 20 6‐km transects from mid‐April 2009 to mid‐May 2010 using the marked nest count method (Kühl, Maisels, Ancrenaz, & Williamson, 2008). We set transects at a 45° bearing, to cross all major rivers (White & Edwards, 2000), and separated from each other by a distance of 300 m (Figure 1). We walked transects every two weeks for 13 months for nest censuses, during which recent night nests (<1‐month‐old) were recorded and marked with red paint to avoid recounting in the next survey. We considered multiple nests to belong to the same nest group when present within a radius of 20 m (gorilla) or 30 m (chimpanzee) (Dupain, Guislain, Nguenang, Vleeschouwer, & Elsacker, 2004; Tagg & Willie, 2013). Because we focused on fresh nests, characteristics, such as the presence of urine, hairs, feces, prints, and feeding remains, helped us distinguish gorilla nests from chimpanzee nests (Sanz, Morgan, Strindberg, & Onononga, 2007). Additionally, along each transect, we noted the habitat type at every 50 m to evaluate habitat availability.

#### 2.2.2 Great ape tracking, fecal sample collection and dietary analysis

From January to December 2014, we collected fecal samples of gorillas and chimpanzees, by tracking one‐day‐old great ape trails to locate fresh nests. We differentiated feces and trails based on the presence of signs such as shape, size, associated odor, hairs (Head, Boesch, Makaga, & Robbins, 2011), sightings, and vocalizations. We washed fecal samples through a 1‐mm sieve and identified extracted seeds to species level where possible, or to genus level (e.g., *Uapaca* spp., *Landolphia *spp., and *Trichoscypha* spp.), and counted them (Doran et al., 2002).

#### 2.2.3 Botanical inventories

We overlaid a grid of 184 500 × 500‐m cells on the area covered by all transects, with one plot of 25 × 25 m at the center of each cell, using ArcGIS 10.3.1 (Figure 1). In each plot, from May 2015 to September 2016, we counted all tree and liana species with DBH ≥10 and ≥5 cm, respectively, that were found in great ape fecal samples. We collected specimens for all plant species that could not be identified on site and sent them for identification at the National Herbarium of Cameroon. In 11 of the plots (6%, *N* = 184), we found no fruiting tree or liana species known to be consumed by gorillas or chimpanzees. We did not collect botanical data from LG as it represents a microhabitat that can occur in any habitat type.

#### 2.2.4 Fruit phenology and fruit characteristics

We selected approximately 10 individuals of each of the fruiting plant species consumed by gorillas and chimpanzees (Djoufack et al., 2007) across 10 of the 6‐km transects, ensuring separation from each other by a distance of at least 600 m, and then measured their DBH and height. We monitored these focal trees monthly (from January 2014 to December 2014) for fruit phenological data. Using a three‐level score (0 = none, 1 = few and 2 = many), we characterized the quantity of fruits seen in the tree or on the ground. We noted information regarding the quantity of unripe fruits and ripe fruits on the tree, and the quantity of unripe and ripe intact fruits on the ground. Each month, we collected random samples of 10 ripe intact fruits from at least three different individuals of each species (Table A1), to measure their weight and count their seeds, to calculate the mean weight and mean number of seeds of each fruiting species.

### 2.3 Statistical analysis

#### 2.3.1 Habitat selection

We compared patterns of habitat selection of gorillas and chimpanzees using the Manly Selectivity Index (Manly, McDonald, Thomas, McDonald, & Erickson, 2002). We used the design 1 sampling protocol, meaning that we considered all nest building observations to have been made at the population level and that we did not identify individuals building nests (Thomas & Taylor, 2006). We then assigned each nest to one habitat type. Equation (1) of the Manly Selectivity Index calculates a selectivity ratio *W_i_* (Manly et al., 2002),

(1) $$W_{i}=\frac{\frac{r_{i}}{p_{i}}}{\sum_{i=1}^{m}\left(\frac{r_{i}}{p_{i}}\right)}$$

(2) $$W_{i}^{\prime }=m\times W_{i}$$

where *r_i_* is the percentage of nests in habitat *i*; *p_i_* is the proportion of habitat *i*, and *m* is the total number of habitat types. The selectivity ratio, *W_i_,* varies on a scale from 0 (avoidance) to 1 (preference). We used the selectivity ratio to calculate a second value, *W'_i_*, (Equation (2) which is used to decide whether a habitat is preferred or used in a proportion less than expected by chance. Values of *W′* <1 indicate that the habitat is used in a proportion less than expected by chance, while values of *W′* >1 indicate a preferred habitat. We applied a chi‐squared goodness‐of‐fit test to quantify the significance of the preference or avoidance status of a habitat type (Neu, Byers, & Peek, 1974). We evaluated patterns of habitat selection using the package *adehabitatHS *(Calenge, 2006) in R version 3.4.2 (R Core Team, 2018).

#### 2.3.2 Preferred and fallback fruits

We used three parameters [Stem density (D), Fruit Availability Potential (FAP), Mean Consumption Score (MCS)] to calculate Global Importance Score (GIS), that in turn was used to classify fruiting plant species into their order of preference by gorillas and chimpanzees, adapting the formulae used in (Doran et al., 2002). D for each species was calculated as follows (Equation (3),

(3) $$D_{i}=\frac{\sum_{k=1}^{K}n_{\mathrm{ik}}}{K\times S}$$

where *n_ik_* is the number of individuals of species *i* in plot *k*, *K* is the total number of plots counted and *S* is the area of each plot (in this case, *S* = 625 m^2^). We used D to calculate FAP, using an adaptation of the formula of Tutin, Ham, White, and Harrison (1997) and Basabose and Yamagiwa (2002) (Equation (4),

(4) $${\text{FAP}}_{\mathrm{ij}}=\left\{\frac{\sum_{n=1}^{N}\left({\text{SS}}_{\mathrm{nij}}\times B_{\mathrm{nij}}\right)}{N_{i}}\right\}\times D_{i}$$

where SS*_nij_*is the sum of fruit scores of the individual *n *of species *i* in month *j*, *B_nij_* is the basal area of the individual *n* of species *i* in month *j*, *N_i_* is the number of monitored individuals of species *i*. Before calculating MCS, we first determined the quantity of fruits (QF) for each species occurring in each fecal sample. For each species, the number of fruits in the sample was 1 if the number of seeds of the species in the sample was less than the species‐specific mean number of seeds per fruit; otherwise, the number of fruits corresponded to the number of seeds in the fecal sample divided by the species‐specific mean number of seeds (for decimal numbers, the higher integer was chosen). For species with uncountable seeds such as *Ficus *spp., we calibrated the number of seeds per fruit and then estimated the number of fruits consumed from seeds found in fecal samples. QF was then the number of fruits multiplied by the species‐specific mean weight. MCS was subsequently calculated using Equation (5),

(5) $${\text{MCS}}_{\mathrm{ij}}=\left(\frac{{\text{QF}}_{\mathrm{ij}}}{\sum_{i=1}^{N_{j}}{\text{QF}}_{\mathrm{ij}}}\right)\times \left(\frac{{\text{Pf}}_{\mathrm{ij}}}{{\text{NPf}}_{j}}\right)$$

where QF*_ij_* is the quantity of fruits of species *i* in month *j*, *N_j_* is the number of species found in fecal samples in month *j*, Pf*_ij_* is the number of fecal samples of month *j* where species *i* was present, and NPf*_j_* is the number of fecal samples analyzed in month *j*. We calculated the GIS, using the FAP and the MCS (Equation (6),

(6) $${\text{GIS}}_{i}=\left\{\frac{\sum_{j=1}^{J}({\text{MCS}}_{\mathrm{ij}}/{\text{FAP}}_{\mathrm{ij}})}{J}\right\}\times {\text{Pe}}_{i}$$

where *J* is the number of months of study, and Pe*_i_* is the proportion of months during which species *i *was consumed, calculated relative to the number of months during which the species was bearing fruit. We classified species based on their GIS value, with higher values corresponding to more preferred species and lower values corresponding to less preferred species.

Our calculation is an improvement of previous methods that were solely based on the percentage of fecal samples in which the fruits were found (Basabose & Yamagiwa, 2002; Etiendem & Tagg, 2013). A classification based on importance types (Doran et al., 2002) was recommended by Rogers et al. (2004); however, our approach can inform on the relative preference of a plant species by two animal consumers, by comparing their GIS values for that plant species. Another advantage of our approach is that the equations consider seasonal variation in fruit availability, and the combination of FAP and MCS facilitates the identification of fallback species.

We used nonmetric multidimensional scaling (NMDS) ordination to visualize how the FAP of each plant species and the MCS of each fruiting plant species by each great ape species respond to seasonal variation, using the Bray–Curtis dissimilarity (Oksanen et al., 2018). We used PERMANOVA to test the hypothesis that the four seasons have different centroids, for FAP and MCS (Anderson & Walsh, 2013). We also tested for homogeneity within seasons to confirm the results of the PERMANOVA (Oksanen et al., 2018).

We determined fallback fruiting plant species for gorillas and chimpanzees by considering the trend of the FAP of each plant species between seasons, and the MCS of each species, for gorillas and chimpanzees (Harrison & Marshall, 2011; Yamagiwa & Basabose, 2009). We divided the seasons into two groups: two seasons of higher total FAP and two seasons of lower FAP. We considered a species fallback for either gorillas or chimpanzees if it fruited in at least three seasons, and if the trend of MCS was negative to that of the FAP. Then, we considered as fallback those fruiting species with high FAP in seasons of high fruit availability, but with high MCS in a season of lower fruit availability. We produced the respective lists of preferred and fallback species for both gorillas and chimpanzees as follows: for each great ape species, we divided the number of fruiting plant species by 3. We started selecting fallback species before selecting preferred ones (Harrison & Marshall, 2011); this means that a species that could be classified as both preferred and fallback was considered fallback. We ran NMDS, PERMANOVA, and the homogeneity test using the package *vegan* (Oksanen et al., 2018) in R version 3.4.2 (R Core Team, 2018). The role of ordination is to synthesize multivariate data into a fewer number of dimensions (axes) to facilitate the interpretation by displaying the results graphically and the first two axes are generally used (Syms, 2008).

#### 2.3.3 Distribution of Plant consumption traits

We defined the plant consumption traits as: preferred by either gorillas or chimpanzees (“Preferred chimpanzee” and “Preferred gorilla”), preferred by both gorillas and chimpanzees (“Preferred apes”), fallback for either gorillas or chimpanzees (“Fallback chimpanzee” and “Fallback gorilla”), or fallback for both gorillas and chimpanzees (“Fallback apes”). We used Correspondence Analysis (CA) to analyze the multivariate data (Hill, 1974) of species traits, as proposed by Pla, Casanoves, and Rienzo (2012). CA is an indirect gradient ordination based on weighted averaging, which uses the position of the sample to identify that of species (or consumption traits in our case), and vice versa (Lepš & Šmilauer, 2003). In CA, we calculated deviations from expected frequencies so as to have a mean weight of zero, and scores are chosen in a way that minimizes the correlation between rows and columns (traits and habitats) (Hill, 1974). CA is more accurate when the number of species is small (Fayolle et al., 2014) and is, therefore, suitable for the limited number of plant traits and species in our study. We used the first two axes to illustrate the divergence of plant traits and plant species abundance across the different habitat types. The CA results are based on the hypothesis of independence between habitat types and fruiting plant traits (Casanoves, Chapman, & Wrangham, 2012); it then calculates a matrix of chi‐squared deviation that measures the combination of habitat types and plant traits that have more inertia and that contribute to the rejection of the null hypothesis (Casanoves et al., 1995). Finally, CA also provides a row–column sum to zero contingency table, which represents coefficients of the relationship between the two variables in consideration. In this case, this table shows for each plant consumption trait or species, the habitat in which it has its highest abundance; and for each habitat type, with which plant consumption traits or species it is more closely associated. We excluded the 11 plots where no great ape consumed fruiting plant species were found before running the analysis. We used the software package *Infostat 2016* to conduct the CA (Casanoves et al., 1995).

## 3 RESULTS

Tables A2 and A3 present the number of nests and fecal samples collected per species per months, respectively.

### 3.1 Seasonal habitat selection by gorillas and chimpanzees

**Figure 2:**
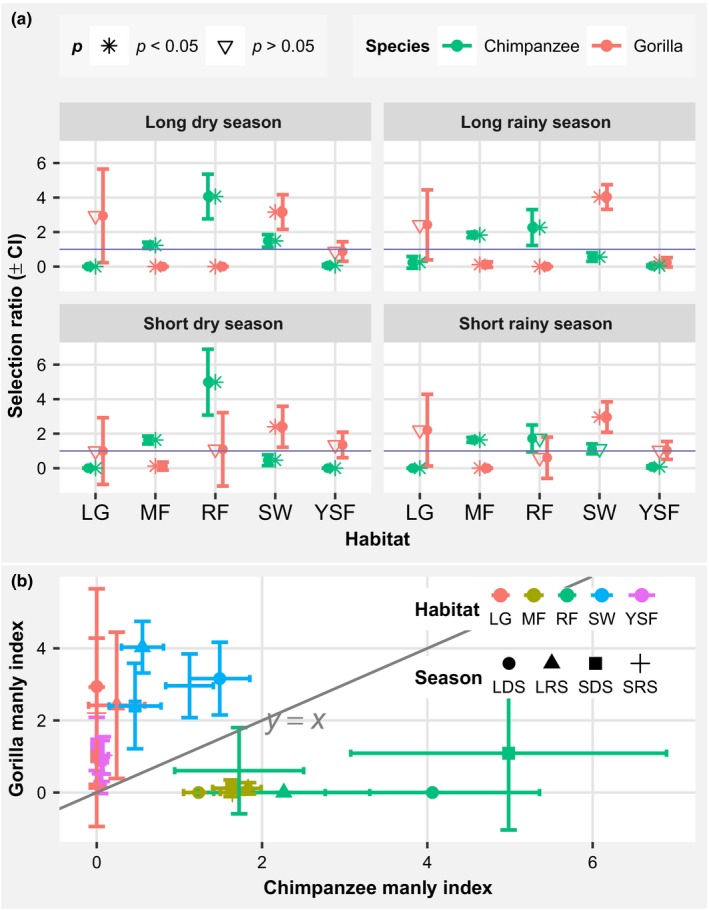
Habitat selection by gorillas and chimpanzees: (a) seasonal variation in habitat selection, habitats with selection ratio >1 are significantly selected and those with selection ratio <1 are significantly avoided; (b) relationship between gorilla and chimpanzee habitat selection indexes, habitat points above the oblique line represent the use by gorillas and those under the line represent the use by chimpanzees. LG: Light Gap; MF: mature forest; RF: riparian forest; YSF: young secondary forest, SW: Swamp

**Figure A2:**
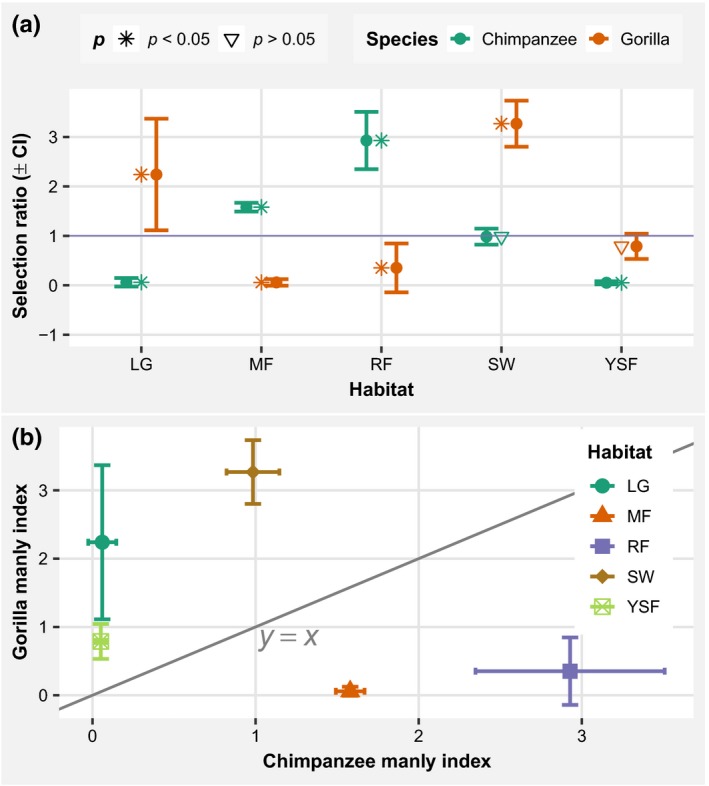
Habitat selection by gorillas and chimpanzees: (a) habitat selection, habitats with selection ratio >1 are significantly selected and those with selection ratio <1 are significantly avoided; (b) relationship between gorilla and chimpanzee habitat selection indexes, habitat points above the oblique line represent the use by gorillas and those under the line represent the use by chimpanzees. LG: Light Gap; MF: mature forest; RF: riparian forest; YSF: young secondary forest, SW: Swamp

The selection of habitats by both gorillas and chimpanzees in all seasons was significantly different to habitat availability (Table 1). Gorillas significantly preferred nesting in SW in all seasons and nested in MF in proportion significantly less than would be expected by chance in all seasons (Figure 2a). For gorillas, the use of LG was proportional to its availability in all seasons and the use of YSF was proportional to its availability in the long dry, short dry and short rainy seasons, but in proportion significantly less than would be expected by chance in the long rainy season (Figure 2a). Also, RF was used proportionally to its availability by gorillas in the short dry and short rainy seasons but was in proportion significantly less than would be expected by chance in the long dry and long rainy seasons (Figure 2a). Chimpanzees significantly preferred nesting in MF in all seasons, and significantly nested in LG and YSF less than would be expected by chance in all seasons (Figure 2a). Chimpanzees significantly nested in SW in proportion significantly less than would be expected by chance in the short dry and long rainy seasons, used it proportionally to its availability in the short rainy season, and significantly preferred it in the long dry season (Figure 2a). Overall, chimpanzees and gorillas both preferentially used different sets of habitat types: gorillas commonly used LG, SW, and YSF, while chimpanzees commonly used MF and RF (Figure 2b). Gorillas very rarely used MF, while chimpanzees very rarely used LG and YSF (Figure 2b). Gorillas seasonally increased their use of RF (in the short dry season), while chimpanzees seasonally increased their use of SW (in the long dry season) (Figure 2b), which are not their preferred habitats. In the analysis of all seasons pooled in the present study, we found light gaps to be significantly preferred by gorillas, and YSF to remain used proportionally to its availability (Figure A2a,b). However, the present study reveals a use of LG by gorillas across all seasons proportionally to its availability, but the mean selectivity indexes are greater than 1 in all seasons (Figure 2a). This nonsignificance may be due to the low number of data points of nests recorded in that habitat type, which would also explain the longer error bars in all seasons for light gaps (Figure 2a).

**Table 1:** Chi‐square of Manly Selectivity test for habitat use by gorillas and chimpanzees in the different seasons

Season	Chimpanzee			Gorilla		
	Chi‐square	*df*	*p*‐value	Chi‐square	*df*	*p*‐value
Long dry	148.167	4	0.000	43.554	4	0.000
Short dry	116.874	4	0.000	16.441	4	0.002
Long rainy	141.601	4	0.000	78.267	4	0.000
Short rainy	160.201	4	0.000	49.441	4	0.000

### 3.2 Preferred and fallback fruits for gorillas and chimpanzees

**Figure 3:**
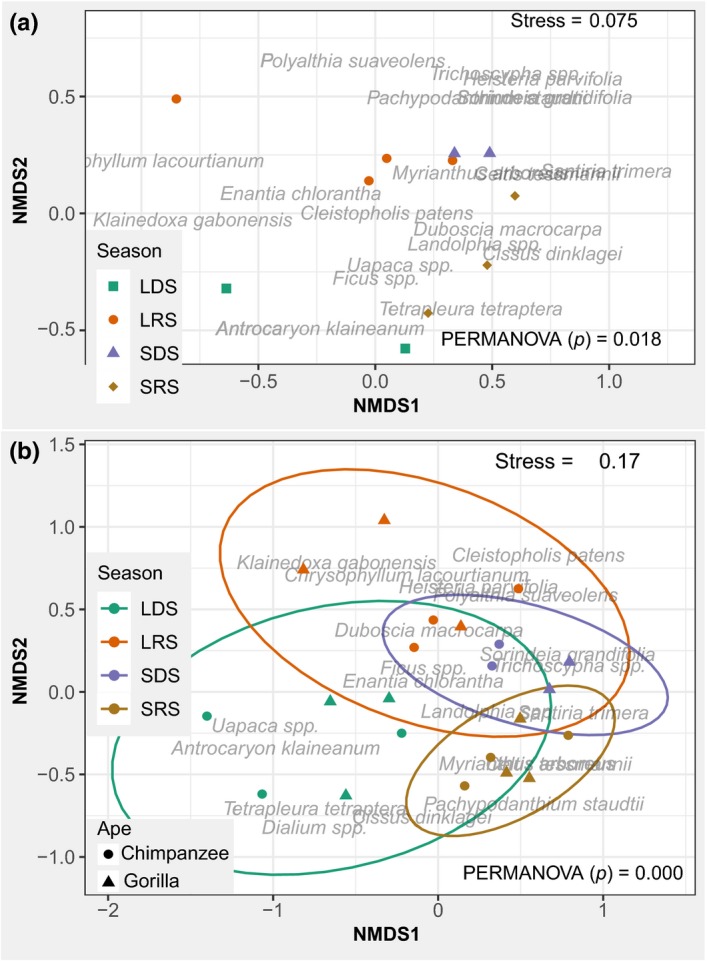
NMDS ordination results depicting FAP fruit availability potential (a) and MCS Mean consumption score (b) in relation to seasons, LDS: long dry season, LRS: long rainy season, SDS: short rainy season, and SRS: short rainy season. The closer the points (months), the more similar they are in terms of: the plant species bearing fruits as well as their corresponding FAP (a), the plant species consumed as well as their MCS (b)

We used NMDS to evaluate whether FAP and MCS vary between seasons (Figure 3a,b). The stress values of the two plots are lower than 0.2, indicating that the two axes easily represent the configuration of the data (Quinn & Keough, 2002). Neither axis 1 nor 2 separates the diversity of FAP (Figure 3a) or MCS (Figure 3b) between seasons, but there seems to exist a separation between seasons. The PERMANOVA results were significant for both MCS and FAP, confirming the observed differences (Figure 3a,b). Additionally, we obtained a nonsignificant within‐season dispersion (*p* = 0.346 for MCS, and *p* = 0.370 for FAP), indicating a confidence in our PERMANOVA results. Meanwhile, it is notable that the seasonal variation in fruit consumption by gorillas and chimpanzees follows the same pattern, as approximately the same species were consumed by both gorillas and chimpanzees in all seasons (Figure 3b). Also, the ordination of MCS (Figure 3b) presents approximately the same configuration as that of FAP (Figure 3a), meaning that the MCS of many plant species for gorillas and chimpanzees vary according to their FAP. An exception is *Uapaca *spp., which produces fruits in almost all seasons, but was highly consumed by chimpanzees more than gorillas in the long dry season (Figure 3a,b). Similarly, *Chrysophyllum lacourtianum *and *Klainedoxa gabonensis* in the long rainy season were consumed more by gorillas (Figure 3b). Many species, such as *Trichoscypha *spp., *Sorindeia grandifolia* and *Santiria trimera* were more available in the short dry season and were highly consumed in that season, almost exclusively by chimpanzees (Figure 3a,b).

**Figure 4:**
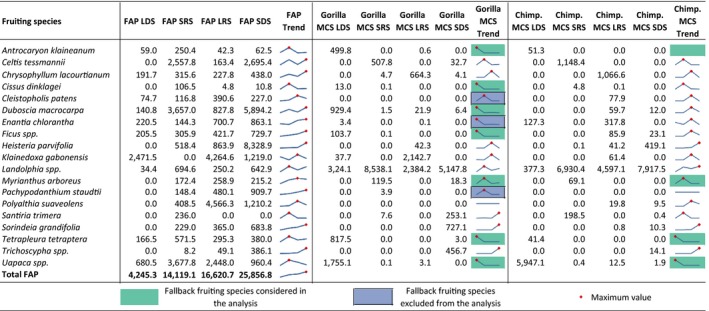
Fallback fruit species determination. Lines present the trends of FAP and MCS; *FAP*: fruit availability potential, MCS: mean consumption score, LDS: long dry season, LRS: long rainy season, SDS: short rainy season, SRS: short rainy season, and Chimp.: Chimpanzee. Excluded species are with too low MCS values

Gorillas and chimpanzees did not exhibit the same order of preference for fruits (Table 2). *Landolphia *spp. fruits are highly preferred by both great ape species, but many other species highly preferred by chimpanzees (namely *Santiria trimera*, *Enantia chlorantha,* and *Celtis tessmannii*) are less preferred by gorillas. Similarly, *Ficus *spp., *Tetrapleura tetraptera,* and *Sorindeia grandifolia* are highly preferred by gorillas and less preferred by chimpanzees (Table 2). Regarding fallback fruits, many species were consumed by both gorillas and chimpanzees in inverse proportion to the overall trend in fruit availability (Figure 4). Then, *Antrocaryon klaineanum*, *Myrianthus arboreus*, *Tetrapleura tetraptera,* and *Uapaca *spp. are fallback fruits for both gorillas and chimpanzees, while *Ficus *spp., *Cissus dinklagei,* and *Duboscia macrocarpa* are fallback species for gorillas but not for chimpanzees (Figure 4). Because we first selected fallback species before selecting for preferred ones, a highly preferred species (e.g., *Uapaca *spp.), from our calculations, was classified as a fallback (Table 2, Figure 4). We attributed a species to either “fallback” or “preferred” using different criteria. Then it is likely that some species may appear in both categories. But in our case, we needed to assign a species to only one category before proceeding the analyses as recommended by Harrison and Marshall (2011). We found that four species (*Ficus *spp., *Uapaca *spp., *Tetrapleura tetraptera*, *Antrocaryon klaineanum*) for gorillas and two species (*Uapaca *spp., *Antrocaryon klaineanum*) for chimpanzees were classified as both fallback and preferred and were then assigned to fallback.

**Table 2:** Fruit preference orders for gorillas and chimpanzees

Species name	Family	Chimpanzee		Gorilla	
		GIS	Rank	GIS	Rank
*Landolphia *spp.^a^	Apocynaceae	3285.339	1#	608.751	1#
*Chrysophyllum lacourtianum*	Sapotaceae	60.898	2#	17.899	4#
*Santiria trimera*	Burseraceae	22.911	3#	0.883	11#
*Uapaca *spp.^b^	Euphorbiaceae	16.803	4##	10.861	6##
*Enantia chlorantha*	Annonaceae	3.306	5#	0.053	14
*Celtis tessmannii*	Ulmaceae	3.276	6#	2.086	10#
*Antrocaryon klaineanum*	Anacardiaceae	1.581	7##	91.494	2##
*Heisteria parvifolia*	Olacaceae	1.554	8#	0.122	13
*Cleistopholis patens*	Annonacae	1.350	9#	0^e^	**17**
*Ficus *spp.^c^	Moraceae	0.935	10	27.492	3##
*Duboscia macrocarpa*	Tiliaceae	0.835	11	0.461	12##
*Cissus dinklagei*	Vitaceae	0.517	12	0.011	15##
*Tetrapleura tetraptera*	Mimosaceae	0.251	13##	9.947	7##
*Trichoscypha *spp.^d^	Anacardiaceae	0.178	14	8.014	8#
*Sorindeia grandifolia*	Anacardiaceae	0.175	15	13.175	5#
*Polyalthia suaveolens*	Annonaceae	0.050	16	*	**UC**
*Klainedoxa gabonensis*	Irvingiaceae	0.031	17	4.835	9#
*Myrianthus arboreus*	Urticaceae	* *	**UC** ##	0.004	16##
*Pachypodanthium staudtii*	Annonaceae	* *	**UC**	* *	**UC**
*Dialium *spp.	Ceasalpiniaceae	* * *	**UC**	* * *	**UC**

Note

GIS: Global Importance Score; UC: unclassified; ^e^: GIS <0.001.

a Includes* L. glabra, L. jumellei, L. landolphioides, L. mannii, L. maxima, L. owariensis, L. violacea, L. jumellei*, and two unidentified species.

b Includes *U. acuminata, U. guineensis, U. paludosa, U. vanhoutei*.

c Includes *Ficus mucuso*, and some stranglers.

d Includes *T. abut *and *T. acuminata*.

* Not consumed.

** Consumed but not found during phenological surveys.

*** consumed but not found neither in phenological surveys nor in botanical inventories.

^#^ Preferred species.

^##^ Fallback species.

### 3.3 Spatial distribution of fruiting plants in relation to their consumption traits

**Figure 5:**
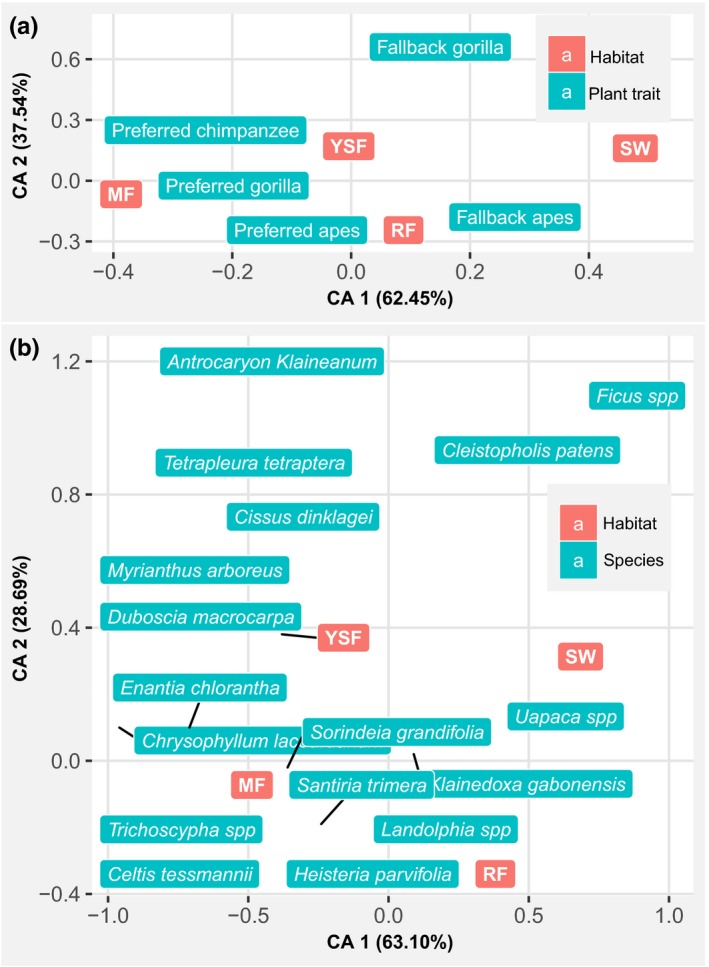
Results of the Correspondence Analysis showing the distribution of fruiting species and their consumption traits in different habitat types: (a) plant consumption traits; (b) individual species. MF: mature forest; RF: riparian forest; YSF: young secondary forest; SW: Swamp. The black lines indicate the exact location of the labels to which they are linked and are used to avoid overlap of several labels at the same location; the percentages represent the relative quantity of inertia “extracted”

**Figure 6:**
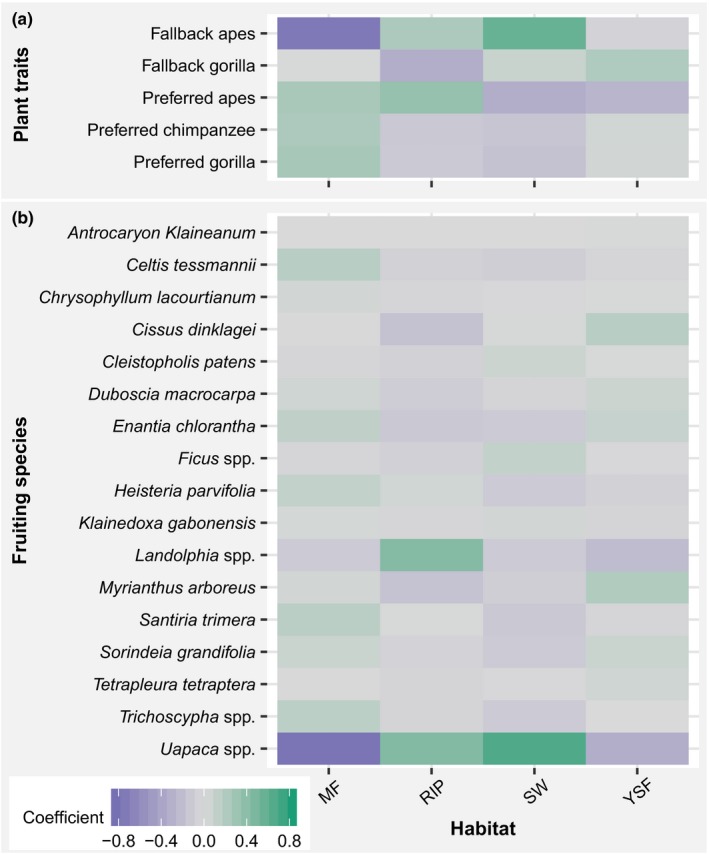
Relationship between plant consumption traits and individual species and habitat types: (a) plant consumption traits; (b) individual species. MF: mature forest; RFL riparian forest; YSF: young secondary forest; SW: Swamp. The data used in this graph are from the row–column sum to zero contingency tables of the Correspondence Analyses

The results of CA indicate a high correlation between MF and SW and the first axis, while YSF and RF are weakly correlated with the first axis (Figure 5a). Additionally, YSF and RF have their highest correlation with the second axis, but YSF and SW have a similar correlation with the second axis (Figure 5a). MF and SW are farther from the center and are located on both sides of the center, meaning that these habitat types contribute the most to the differences in the distribution of plant consumption traits (Figure 5a) and species (Figure 5b) across all habitats. Their different correlation signs (the projection of each object to the axis) to the first axis mean that the abundances of plant consumption traits (Figure 5a) and species (Figure 5b) are different between those two habitats. The proximity of YSF and RF to the center on the first axis indicates that they weakly contribute to the separation of the plant consumption traits. The ordination plot has separated two plant consumption traits categories: the group of preferred plant species, negatively correlated with the first axis as MF, and fallback species positively correlated with the first axis as SW (Figure 5a). The first two axes of the ordination plots (Figure 5a,b) explained the high percentages of total variability (99.99% for plant consumption traits [Figure 5a] and 91.79% for individual species [Figure 5b]). The same pattern of correlation between habitat types and ordination axis for plant consumption traits was observed for individual species (Figure 5a,b). The highest abundances of most species are shifted toward MF, YSF, and RF, while just a few sets of species are associated with SW, indicating that the abundance of fewer species may influence the association of plant traits to habitat types (Figure 5). Under the assumption of independence between variables, the relationship between plant traits and individual species reveals that fallback fruiting species of gorillas alone, and those of both gorillas and chimpanzees are the most indicating plant traits (Figure 6a), meaning that they are more associated with some habitat types than other plant traits. As indicated by the ordination plot (Figure 5a), the strongest discriminating nature of MF and SW is due to the distribution of fallback species common to both gorillas and chimpanzees, that are highly abundant in SW, but almost absent from MF (Figure 6a). The trait of fallback species for gorillas is more abundant in YSF; while that of preferred species of chimpanzees and of gorillas are positively associated with both RF and MF (Figure 6a). All strictly preferred plant traits are only positively associated with MF. The abundance of *Uapaca *spp. is the most influenced by habitat types, with the highest values in SW, and may be responsible for the high abundance of fallback fruits for both gorillas and chimpanzees in SW (Figure 6a,b). *Myrianthus arboreus*, *Landolphia *spp., *Celtis tessmanii,* and *Santiria trimera* are also highly differentiated, with their highest abundance respectively in YSF and RF for the first two, and MF for the rest (Figure 6b). Many species, such as *Antrocaryon klaineanum*, *Chrysophyllum lacourtianum,* and *Klainedoxa gabonensis,* do not relatively show any association pattern with habitat types (Figure 6b). They certainly influence less the abundances of plant consumption traits by habitat types (Figure 6a).

It should be noted that *Uapaca *spp. is the only fruiting plant species accounting for the high abundance of fallback fruits to both gorillas and chimpanzees (fallback apes) in swamps (Figure 6a,b). Given that this species was mostly consumed in the long dry season by gorillas and chimpanzees, with chimpanzees consuming more than gorillas (Figure 3b), and that *Uapaca *spp. is the most abundant great ape fruiting plant species found in swamps (Figure 5b), this may explain why chimpanzees significantly selected swamps for nest building in the long dry season (Figure 2a). Furthermore, *Landolphia *spp. was found to be highly preferred by both gorillas and chimpanzees and is the principal determinant of the higher abundance of joint preferred fruiting species (preferred apes) in riparian forests (Figure 6). Its consumption by gorillas and chimpanzees was higher in the short dry and short rainy seasons (Figure 3b), possibly explaining the slight increase in gorilla nests found in riparian forests during the short dry and short rainy seasons (Figure 2b).

## 4 DISCUSSION

Resource competition (interference and exploitation) is an important factor structuring ecological communities (Pianka, 1981). In these cases, coexistence may only be possible if species use different habitat types or resources, or if they partition shared resources, according to their temporal availability or spatial distribution (Amarasekare, 2003). The results of this study indicate that gorillas and chimpanzees preferred different sets of habitat types, but seasonal variation in the pattern of habitat use was observed, thus not supporting the hypothesis that the pattern of habitat use promotes coexistence of gorillas and chimpanzees. We also found that gorilla and chimpanzee preferred and fallback fruiting species were different, but due to the nonrandom pattern of habitat use, this does not support the hypothesis of dietary niche separation as a mechanism of coexistence of gorillas and chimpanzees. However, considering the differential distribution of fruiting woody plant consumption traits, we found that chimpanzees may prefer habitat types where their preferred fruiting plant species are more abundant, while gorillas may prefer habitat types where their fallback fruiting species are more abundant, thus supporting the hypothesis of interaction between two niche axes (dietary and habitat niche) in the promotion of coexistence of gorillas and chimpanzees.

The main limitation of our study is that we used data on great ape habitat use and fruit consumption collected in different years. Due to the inter‐annual variability in fruit phenology, the results of fruit preference and plant consumption traits distribution may not reflect exactly the pattern of habitat use when nest surveys were conducted. However, we still consider our results to be reliable because similar patterns of fruiting periods and FAP, fruit preference and fallback were observed in other sites for several common plant species (Doran et al., 2002; Doran‐Sheehy et al., 2009; Harrison & Marshall, 2011; Head et al., 2011; Nishihara, 1995; Remis, 1997; Rogers, Voysey, McDonald, Parnell, & Tutin, 1998), and through previous research in our research site (Deblauwe, 2009; Petre, 2016). We consider our results of fruit availability and fruit consumption by sympatric gorillas and chimpanzees to reflect a general pattern that can be used to explain other aspects of their ecology in the same site. An additional limitation is that the patterns of habitat use in this study are based only on nesting data because we did not collect data from other signs, such as footprints, feeding remains and vocalizations, to evaluate the pattern of habitat use although our mechanism of coexistence suggests a possible movement of ape species between habitat types. Our interpretations are based on the results of Furuichi, Hashimoto, and Tashiro (2001) and Morgan et al. (2006) that great apes range in habitats that are not their preferred nesting habitats.

We calculated the FAP using fruiting scores rather than the true quantity of fruits in the trees; however, our results are still useful in that our FAP provides a relative fruit quantity as the calculation integrates the DBH. Studies have considered the “fallen fruit phenology” method to quantify fruit availability (Chapman, Wrangham, & Chapman, 1994). But the “fallen fruit phenology” method has a disadvantage that it misses all species whose fruits do not fall on the ground or species whose fruits are consumed by animals before they fall on the ground (Takenoshita, Ando, Iwata, & Yamagiwa, 2008). We then considered the method based on fruit observation in trees to be more appropriate in order to provide an estimation for all species.

### 4.1 Seasonal change in patterns of habitat use and great ape coexistence

We found seasonal variation in habitat use by both gorillas and chimpanzees. We noted that both species consistently preferred or avoided certain habitats but that there was also a seasonal increase in the selection index of other habitat types. Our results report a niche partitioning based on habitat use between gorillas (preferring swamps) and chimpanzees (preferring mature forests), except in the long dry season when both species significantly preferred the same habitat (swamps). Niche partitioning via differential habitat selection by gorillas and chimpanzees has been shown in previous studies (Arnhem et al., 2008; Morgan et al., 2006). Patterns of habitat use by gorillas and chimpanzees observed in other studies have shown chimpanzees to preferentially nest in mature forests and gorillas in swamps, open canopy forests, and young secondary forests (Bermejo, 1999; Fay & Agnagna, 1992; Ogawa, Yoshikawa, & Idani, 2014; Rainey et al., 2010; Willie et al., 2013). However, our results are not consistent with these observations. Our seasonal analysis revealed a significant increase in swamp use by chimpanzees in the long dry season, as observed elsewhere (Morgan et al., 2006; Poulsen & Clark, 2004) and in the same site in a previous study (Tagg, Willie, Petre, & Haggis, 2013). We found gorillas to randomly use LG and YSF in all seasons, except in the long rainy season when they used YSF in proportion significantly less than would be expected by chance; however, a general analysis revealed that gorillas preferred LG, but again used YSF in proportion significantly less than would be expected by chance. A recent study in the same region but using a different data set also found a general use of YSF proportionally to its availability and a preference of LG by gorillas and subsequently considered young secondary forests to be an important habitat for gorilla survival because of the higher percentage of nests built (Tédonzong et al., 2018).

Due to the spatial heterogeneity, gorillas and chimpanzees may have reached a stable local coexistence across their range on the basis of general habitat partitioning (Amarasekare, 2009; Ritchie, 2002). However, our results showed a significant increase in swamp use in the long dry and short rainy seasons relative to the short dry and long rainy seasons by chimpanzees, and an increase in the use of riparian forests from significantly avoided in the long dry and long rainy seasons to randomly used in the short dry and short rainy seasons by gorillas (Figure 2a). This suggests that the niches of the two great ape species may overlap in swamps in the dry season and therefore does not support the suggestion that coexistence is enabled as a result of differential habitat use. An explanation that supports the increased use of swamps by chimpanzees in the long dry season is that chimpanzees do so to avoid hunters (Dupain et al., 2004; Kalan, Madzoké, & Rainey, 2010; Poulsen & Clark, 2004; Willie et al., 2013), in contrast to the proposition that mature forests may constitute a refuge for chimpanzees (Ogawa et al., 2014).

It is possible that chimpanzee nesting patterns may be influenced by the presence of gorillas (Head, Robbins, Mundry, Makaga, and Boesch (2012) and may be linked to the seasonal availability of fruits (Head et al., 2012). Our results support this, as they reveal patterns of habitat segregation between chimpanzees and gorillas in different habitat types and seasons, therefore suggesting competitive avoidance between gorillas and chimpanzees. However, patterns of habitat separation may be biased if we focus our analysis only on nests. A combination of direct (sightings, vocalizations, etc.) and indirect (nest surveys, etc.) observations reveal different patterns of habitat use for both gorillas and chimpanzees being obtained depending on the method used (Furuichi et al., 2001; Morgan et al., 2006). Although the pattern of habitat selection by chimpanzees estimated via nest counts reflected that obtained through direct observations (Furuichi et al., 2001), patterns of habitat preference by gorillas estimated via nest counts were not observed through direct observation (Morgan et al., 2006). This dichotomy in habitat use patterns based on methods of estimation coupled with seasonal variation in habitat selection indicates that other factors may be responsible for the coexistence of gorillas and chimpanzees. Foraging is a principal determinant of habitat use, and as frugivores, the seasonal nature of fruit availability may impact the seasonal patterns of habitat use by gorillas and chimpanzees (Basabose, 2005; Poulsen & Clark, 2004). Then examining the patterns of fruit seasonal partitioning between gorillas and chimpanzees may provide additional insights into their coexistence.

### 4.2 Fruit preference, fallback fruits, and coexistence of gorillas and chimpanzees

Coexistence between great apes may be possible due to differences in fruit consumption (Morgan & Sanz, 2006; Schreier, Harcourt, Coppeto, & Somi, 2009; Vleut, Galindo‐González, Boer, Levy‐Tacher, & Vazquez, 2015). Our results indicate that gorillas and chimpanzees consumed almost the same fruiting plant species, which is consistent with the high overlap in fruit consumption observed in other sites (Basabose & Yamagiwa, 2002; Oelze et al., 2014; Tutin et al., 1991). Although gorillas and chimpanzees consume a similar array of fruiting species, they may differ in their seasonal changes in the MCS of different species. Fruits from *Dialium *spp. were found in fecal samples but were not found in botanical inventories (Table 1), maybe because its density is very low in the site. A study conducted in an adjacent site in the Dja Reserve reported several species of *Dialium *spp. (Sonké & Couvreur, 2014); however, we cannot confirm the presence of *Dialium* spp. in the present study site. If the species is not present, great apes may be migrating beyond the study area to consume fruits from *Dialium* spp. However, the high consumption of *Dialium* spp. fruits in the dry season, when fruit availability is low, was also observed by Masi and Breuer (2018) in the Republic of Congo and in the Central African Republic.

Chimpanzees and gorillas also exhibited a differing order of preference for fruiting plants (Table 2). In addition, as well as there being fruiting species used by gorillas and chimpanzees as fallback fruits, many other fruits are fallback for gorillas alone (Figure 4). Dietary niche partitioning between gorillas and chimpanzees has long been viewed at the level of diet guilds, considering fruits as preferred and herbaceous and other vegetative foods as fallback (Doran‐Sheehy, Mongo, Lodwick, & Conklin‐Brittain, 2008; Doran‐Sheehy et al., 2009; Rogers et al., 2004; Williamson, Tutin, Rogers, & Fernandez, 1990). Although fallback foods have been viewed as those of low nutritional value (Doran‐Sheehy et al., 2008; Rogers et al., 2004), determination of fallback foods has rarely been carried out at the species level (Basabose & Yamagiwa, 2002). However, the protein content of fruits may be an important source of energy for great apes (Felton et al., 2009), and the nutritional content of fruiting species may differ (Masi et al., 2015). Our results support the classification by many other studies of *Ficus *spp. as a fallback fruit for chimpanzees (Harrison & Marshall, 2011) and a preferred food for gorillas (Chapman, Chapman, Zanne, Poulsen, & Clark, 2005; Yamagiwa & Basabose, 2009). Our results also show that both gorillas and chimpanzees considerably increased their consumption of *Uapaca *spp. fruits in low fruit availability seasons (Figure 4). *Landolphia *spp. fruits were mostly available in the short dry, short rainy and long rainy seasons, and in those seasons they were highly incorporated into the diet of both great ape species (Figure 4). Head et al. (2011) classified *Uapaca* spp. as one of the top 10 most consumed species by gorillas but not for chimpanzees, based on the frequency of fecal samples containing their seeds. Because this classification was only based on the frequency of consumption but did not consider the quantity consumed and the availability, this may explain why in our study *Uapaca* spp. fruits were classified as fallback and preferred for both gorillas and chimpanzees. The quantity of fruit consumed may increase the MCS and then the final GIS, but do not change the fact that the species is highly consumed when other fruiting species are scarce. Likewise, as shown by Felton et al. (2009) and Masi, Cipolletta, Ortmann, Mundry, and Robbins (2009), gorillas and chimpanzees may maintain their protein intake consistently, increasing total energy intake by incorporating different food types with different nutritional compositions. This may explain why both gorillas and chimpanzees, while being highly frugivorous, generally incorporate nonfruit foods (herbaceous plants, tree leaves, tree barks) into their diets (Doran & Mcneilage, 1999; Doran et al., 2002; Remis, 1997; Tweheyo & Lye, 2005). Previous studies have found that gorillas shift their diet in response to lower fruit availability to consume nonfruit foods (generally herbaceous plants) while chimpanzees maintain a fruit‐dominated diet (Basabose & Yamagiwa, 2002; Head et al., 2011), and this process may be viewed as a niche partitioning mechanism.

Among the few species found to be important in the chimpanzee diet during low fruit availability periods by Head et al. (2011), it appeared that gorillas never consumed lipid‐rich fruiting species such as *Staudtia gabonensis *and *Pycnanthus angolensis*, in any season; however, we found none of those species to be consumed by great apes in our study. Gorillas have been observed to avoid some fruit types, thus increasing the relative fruit dietary breadth of chimpanzees in terms of fruits (Head et al., 2011). If chimpanzees consume more fatty‐rich fruits than do gorillas, this may contribute to enabling coexistence between the species, as competitive exclusion can be avoided if there exists an exclusivity in the use of certain resources, in addition to other shared resources (Perrin & Kotler, 2005; Ritchie, 2002). However, this is not supported by observations from Lopé in Gabon where gorillas may consume more fruits than chimpanzees and do not reduce their fruit consumption in low fruit availability seasons relative to chimpanzees (Tutin et al., 1991). Indeed, the diversity of fruits consumed by gorillas and chimpanzees may result in sufficiently large fruit niche breadths so that competition is reduced, despite the high fruit dietary niche overlap (Sushma & Singh, 2006). The fission–fusion behavior exhibited by chimpanzees may be influenced by seasonal variation in fruit availability, enabling large groups to divide into smaller subgroups when fruits are scarce (Chapman et al., 1995). Large party size in chimpanzees affords them an increase in dominance over gorillas for access to fruit trees (Basabose & Yamagiwa, 2002; Lehmann & Boesch, 2004), thus suggesting that intraspecific competition among chimpanzees is higher than interspecific competition between gorillas and chimpanzees, enabling their coexistence (Amarasekare, 2009; Lehmann & Boesch, 2004). Meanwhile, chimpanzees may be globally more specialized in fruit consumption than gorillas, and the more dominant species (Lambert & Rothman, 2015; Morgan & Sanz, 2006; Tutin et al., 1997).

A mechanism of coexistence of gorillas and chimpanzees based on fruit food partitioning is plausible. However, it does not help understand differences in habitat use between species and between seasons and thus does not fully explain the mechanism of coexistence of great apes. We, therefore, continue by exploring if the combination of mechanisms of fruit choice and habitat use may lead to additional explanations.

### 4.3 Spatial distribution of preferred and fallback fruits and coexistence of gorillas and chimpanzees

We found that all preferred fruiting species for both gorillas and chimpanzees (preferred apes) were more abundant in mature forest and riparian forest than in other habitat types, while all fallback fruiting species for both great apes (fallback apes) were more abundant in SW and YSF (Figure 6a). As chimpanzees significantly preferred mature forest, this suggests they may prefer nesting in habitats which hold higher abundances of their preferred fruits. Our results, therefore, suggest that great ape patterns of habitat use may be influenced by the difference in abundance of preferred and fallback woody fruiting plants, the phenology of these plant species and the season in which they are most commonly consumed. This suggests a nonlinear competition, where competitors are affected by temporal variation in food availability (Amarasekare, 2009; Amarasekare, Hoopes, Mouquet, & Holyoak, 2004). However, although all gorilla preferred fruiting plants were highly abundant in mature forest, gorilla nests were not found in that habitat in this study. This may be a consequence of the indirect data collection methodologies adopted for this study; studies using direct observational methods have found gorillas to frequently use mature forests and chimpanzees to use habitat types other than mature forest where they preferably build nests (Furuichi et al., 2001; Morgan et al., 2006), suggesting overlap in terms of ranging habitats (Morgan et al., 2006). A potential mechanism of coexistence of gorillas and chimpanzees, therefore, may be driven by the seasonal variation in habitat selection and the seasonal movements between the different habitats (Amarasekare, 2009). As the dominant species, chimpanzees may nest in habitats where preferred woody fruiting plant species are more abundant, while gorillas nest in habitats where preferred fruiting plants are less abundant, but forage in habitats where preferred fruits are more available, for example in mature forest, to reduce competitive exclusion. This mechanism may create a negative density‐dependent effect in great ape habitat use (Amarasekare, 2003). Our results support the evidence that central chimpanzee densities are closely linked to a higher fruit availability as was found in other chimpanzee subspecies (Potts, Watts, & Wrangham, 2011; Stanford & Nkurunungi, 2003). The suggestion that chimpanzee densities are determined by the availability of fruits is supported by Basabose (2005), who observed chimpanzees visiting gorilla preferred nesting habitats to consume their preferred *Ficus *spp. fruits but remaining close to mature forest, their preferred nesting habitat. Furthermore, this dynamic might increase intraspecific competition in chimpanzees (Amarasekare, 2003), as party sizes increase to defend foraging territories (Lehmann & Dunbar, 2009).

This argument, however, does not explain why both gorillas and chimpanzees significantly preferred swamps in the long dry season where *Uapaca *spp., the species with the highest FAP of the season, is more abundant. Competitive exclusion may be avoided if fruit availability is high (Head et al., 2012). This may explain the simultaneous selection of swamps by gorillas and chimpanzees in the long dry season. First, the long dry season is the season of lowest fruit availability, when great apes are expected to show the greatest niche divergence, but the contrary is observed. Second, the availability of *Uapaca *spp. fruits may be sufficiently high in swamps in the long dry season to sustain both apes, as both are observed to increase *Uapaca* spp. consumption in the long dry season (Figure 4). Swamps are with YSF and TA the habitats with the highest densities in herbaceous plants, and those plants are available year‐round (Rainey et al., 2010; Willie et al., 2013). The ranging patterns of western lowland gorillas in SW at Mondika were found to be correlated with the consumption of herbaceous vegetation, and this consumption occurred occasionally when fruit availability in terra firma forests was low (Doran & Greer, 2002). Thus, additionally to the high quantity of *Uapaca* spp. fruits in swamps in the long dry season, competition between gorillas and chimpanzees may be avoided in swamps by the increasing consumption of herbs by gorillas in the long dry season. Meanwhile, it has been found that herbaceous plants suitable for gorilla nest building are less abundant in mature forest than in old secondary forest in this region (Willie et al., 2013), while chimpanzee nesting trees are less abundant in young secondary forest than in mature forest (Tagg et al., 2013). Adding to the lower abundance of chimpanzee preferred fruiting plants in young secondary forest found in this study, this observation may explain the near absence of gorillas and chimpanzees in MF and in YSF, respectively, in all seasons. The year‐round availability of herbaceous plants makes it nonresponsible for the seasonal ranging patterns of great apes across habitats. Coexistence of gorillas and chimpanzees has been suggested to be favored by a difference in nest height for both species, whereby gorillas may prefer nesting on the ground and chimpanzees in trees, to avoid competition (Stanford, 2006). Certainly, it is widely observed that gorillas commonly nest on the ground, using herbaceous vegetation (Willie et al., 2013).

## 5 CONCLUSION

**Figure 7:**
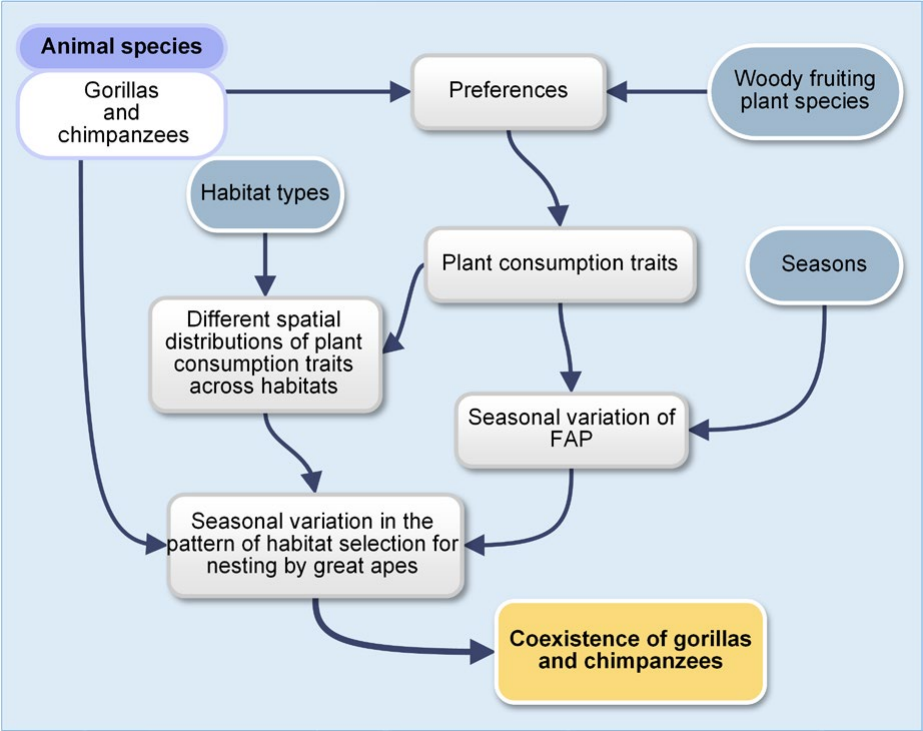
Ecological framework of the coexistence of gorillas and chimpanzees: *FAP* Fruit Availability Potential

This paper addresses how the consumption traits of fruiting woody plant species consumed by sympatric great apes may contribute to shaping their local coexistence. We find that the spatial distribution of fruiting plants according to their extrinsic traits and the temporal availability of fruits for different fruiting woody plant species may account for the coexistence of gorillas and chimpanzees, via a mechanism of seasonal movement between habitats. As predicted, preferred fruiting plants for both gorillas and chimpanzees were most abundant in chimpanzee preferred nesting habitat types, while their fallback fruiting species were more abundant in gorilla preferred nesting habitat types. Tree species may differ in their abundance across habitat types, and the choice of a set of species to be logged will imply different levels of perturbation in different habitat types. This study proposes using the spatial distribution of resources to understand mechanisms of coexistence of two competing species, by defining consumption traits for each fruiting plant consumed, based on its preference and fallback status. Our results conform to the assumption of niche theory. Gorillas and chimpanzees used similar habitat types and fruits, but to avoid competition, they partitioned those resources in space and time. The seasonal nature of fruit availability and the different abundances of the different fruiting plant species of different quality across habitats are two ecological factors that have facilitated niche partitioning between gorillas and chimpanzees (Figure 7). Also, the general tendency of preferred fruiting species for both gorillas and chimpanzees to be more abundant in chimpanzee preferred habitats confirms the competitive superiority of chimpanzees over gorillas. The results of this study can contribute to conservation in human‐modified landscapes in two ways: the findings are helpful in predicting the outcome of environmental change on great ape community structure, and they can be employed in the restoration of degraded habitats.

## CONFLICT OF INTEREST

We have no conflict of interest to declare.

## AUTHOR CONTRIBUTION

All authors conceived the ideas and designed the methodology; LRDT, JW, AMPK, JKK, and CAP led the data collection; LRDT conducted statistical analyses of the data and led the writing of the manuscript. All authors contributed critically to the drafts, approved, and contributed to the final manuscript.

## Data Availability

The botanical, phenological, dietary, and nest survey data supporting our findings can be downloaded on Dryad (Tédonzong et al., 2019).

## Appendix 1

**Table A1:** Number of individual plants monitored for each species for fruit phenology in 2014

Species name	January 2014	February 2014	March 2014	April 2014	May 2014	June 2014	July 2014	August 2014	September 2014	October 2014	November 2014	December 2014
*Antrocaryon Klaineanum*	4	4	2	4	4	4	4	4	4	4	4	4
*Celtis tessmannii*	10	10	9	10	10	10	10	10	10	10	10	10
*Chrysophyllum lacourtianum*	8	8	8	8	8	8	8	8	8	8	8	8
*Cissus dinklagei*	9	9	8	9	9	9	8	8	8	9	9	9
*Cleistopholis patens*	9	9	9	9	8	9	8	8	8	9	9	9
*Duboscia macrocarpa*	19	19	17	19	19	19	19	19	19	19	19	19
*Enantia chlorantha*	11	11	10	11	11	11	11	11	11	11	11	11
*Ficus *spp.	14	14	13	14	14	14	14	14	14	14	14	14
*Heisteria parvifolia*	10	10	10	10	10	10	10	10	10	10	10	10
*Klainedoxa gabonensis*	10	10	9	10	10	10	10	10	10	10	10	10
*Landolphia *spp.	37	37	37	37	36	37	36	36	37	37	37	37
*Myrianthus arboreus*	10	10	9	10	10	10	10	10	10	10	10	10
*Pachypodanthium staudtii*	10	10	9	10	10	10	10	10	10	10	10	10
*Polyalthia suaveolens*	10	10	9	10	10	10	10	10	10	10	10	10
*Santiria trimera*	15	15	14	15	15	15	15	15	15	15	15	15
*Sorindeia grandifolia*	10	10	10	10	10	10	10	10	10	10	10	10
*Tetrapleura tetraptera*	9	9	8	9	9	9	9	9	9	9	9	9
*Trichoscypha *spp.	19	19	17	19	19	19	19	19	19	19	19	19
*Uapaca *spp.	37	37	34	37	37	37	37	37	37	37	37	37
Total	261	261	242	261	259	261	258	258	259	261	261	261

**Table A2:** Number of nests recorded for each animal species, per season, per month, and per habitat

Animal species	Season	Month	Light gap	Near primary forest	Old secondary forest	Riparian forest	Swamp	Young secondary forest	Total
Chimpanzee	Long dry season	December 2009	0	18	17	15	25	3	78
		February 2010	0	15	18	5	6	0	44
		January 2010	0	9	12	12	17	0	50
	Total chimpanzee in the long dry season		0	42	47	32	48	3	172
	Long rainy season	April 2009	0	2	15	0	1	0	18
		April 2010	0	0	2	3	4	0	9
		June 2009	1	20	18	11	4	1	55
		March 2010	0	16	15	0	3	1	35
		May 2009	1	13	25	3	5	0	47
	Total chimpanzee in the long rainy season		2	51	75	17	17	2	164
	Short dry season	August 2009	0	15	17	6	5	0	43
		July 2009	0	19	12	15	3	0	49
	Total chimpanzee in the short dry season		0	34	29		8	0	92
	Short rainy season	November 2009	0	33	25	6	23	0	87
		October 2009	0	51	9	6	12	2	80
		September 2009	0	26	13	6	13	3	61
	Total chimpanzee in the short rainy season		0	110	47	18	48	5	228
Total chimpanzees			2	237	198	67	121	10	656
Gorilla	Long dry season	December 2009	0	0	0	0	1	2	3
		February 2010	0	0	0	0	2	4	6
		January 2010	4	0	0	0	13	1	18
	Total gorilla in the long dry season		4	0	0	0	16	7	27
	Long rainy season	April 2009	0	0	0	0	7	0	7
		April 2010	0	2	0	0	9	0	11
		June 2009	4	0	0	0	4	0	8
		March 2010	1	0	0	0	5	0	6
		May 2009	0	0	0	0	6	3	9
	Total gorilla in the long rainy season		5	2	0	0	31	3	41
	Short dry season	August 2009	1	0	1	0	8	1	11
		July 2009	0	0	0	1	1	7	9
	Total gorilla in the short dry season		1	0	1	1	9	8	20
	Short rainy season	November 2009	1	0	0	0	3	11	15
		October 2009	1	0	0	0	13	0	14
		September 2009	2	0	0	1	4	0	7
	Total gorilla in the short rainy season		4	0	0	1	20	11	36
Total gorillas			14	2	1	2	76	29	124

**Table A3:** Number of fecal samples analyzed for each animal species (gorilla and chimpanzee), per month, and per season in 2014. No fecal sample was collected in March 2014

Season	Month	Chimpanzee	Gorilla	Total
Long dry season	January 2014	53	44	97
	February 2014	4	66	70
	December 2014	19	105	124
	March 2014	0	0	0
Long rainy season	April 2014	9	8	17
	May 2014	7	25	32
	June 2014	98	58	156
Short dry season	July 2014	105	97	202
	August 2014	51	129	180
Short rainy season	September 2014	6	65	71
	October 2014	43	261	304
	November 2014	14	47	61
Total		409	905	1,314
